# Combining APR-246 and HDAC-Inhibitors: A Novel Targeted Treatment Option for Neuroblastoma

**DOI:** 10.3390/cancers13174476

**Published:** 2021-09-05

**Authors:** Michael Müller, Lisa Rösch, Sara Najafi, Charlotte Gatzweiler, Johannes Ridinger, Xenia F. Gerloff, David T. W. Jones, Jochen Baßler, Sina Kreth, Sabine Stainczyk, Karen Frese, Benjamin Meder, Frank Westermann, Till Milde, Heike Peterziel, Olaf Witt, Ina Oehme

**Affiliations:** 1 Hopp Children’s Cancer Center Heidelberg (KiTZ), 69120 Heidelberg, Germany; mic.mueller@kitz-heidelberg.de (M.M.); l.roesch@kitz-heidelberg.de (L.R.); s.najafi@kitz-heidelberg.de (S.N.); charlotte.gatzweiler@kitz-heidelberg.de (C.G.); j.ridinger@kitz-heidelberg.de (J.R.); xenia.gerloff@dkfz-heidelberg.de (X.F.G.); david.jones@kitz-heidelberg.de (D.T.W.J.); s.kreth@kitz-heidelberg.de (S.K.); s.stainczyk@kitz-heidelberg.de (S.S.); f.westermann@kitz-heidelberg.de (F.W.); t.milde@kitz-heidelberg.de (T.M.); h.peterziel@kitz-heidelberg.de (H.P.); o.witt@kitz-heidelberg.de (O.W.); 2 Clinical Cooperation Unit Pediatric Oncology, German Cancer Consortium (DKTK), German Cancer Research Center (DKFZ), 69120 Heidelberg, Germany; 3 Faculty of Medicine, Heidelberg University, 69120 Heidelberg, Germany; 4 Faculty of Biosciences, Heidelberg University, 69120 Heidelberg, Germany; 5 Department of Pediatric Oncology, Hematology and Immunology, University of Heidelberg Medical Center, 69120 Heidelberg, Germany; 6 Pediatric Glioma Research Group, German Cancer Research Center (DKFZ) and German Consortium for Translational Cancer Research (DKTK), 69120 Heidelberg, Germany; 7 Biochemistry Center, Heidelberg University, 69120 Heidelberg, Germany; jochen.bassler@bzh.uni-heidelberg.de; 8 Neuroblastoma Genomics, German Cancer Research Center (DKFZ), 69120 Heidelberg, Germany; 9 Institute for Cardiomyopathies Heidelberg, Heidelberg University, 69120 Heidelberg, Germany; karen.frese@med.uni-heidelberg.de (K.F.); Benjamin.Meder@med.uni-heidelberg.de (B.M.); 10 Genome Technology Center, Stanford University, Stanford, CA 94304, USA

**Keywords:** histone deacetylases, ROS, TP53, small molecule inhibitors, pediatric tumors of the nervous system, precision medicine, response prediction biomarker

## Abstract

**Simple Summary:**

Preclinical analyses identified APR-246 as a potent treatment option for neuroblastoma. However, a specific mode of action, sufficient biomarkers and promising combination partners are still missing. Here, we analyze the susceptibilities of different entities and relate them to gene expression profiles and previously described biomarkers. We propose a gene signature, consisting of 13 genes, as a novel predictive biomarker. Furthermore, we provide evidence that APR-246 directly targets metabolic weaknesses in neuroblastoma cell lines, thus hampering ROS detoxification. This makes APR-246 suitable to be combined with ROS-inducing HDAC inhibitors, a treatment combination that has not been described for neuroblastoma thus far.

**Abstract:**

APR-246 (Eprenetapopt/PRIMA-1^Met^) is a very potent anti-cancer drug in clinical trials and was initially developed as a p53 refolding agent. As an alternative mode of action, the elevation of reactive oxygen species (ROS) has been proposed. Through an in silico analysis, we investigated the responses of approximately 800 cancer cell lines (50 entities; Cancer Therapeutics Response Portal, CTRP) to APR-246 treatment. In particular, neuroblastoma, lymphoma and acute lymphocytic leukemia cells were highly responsive. With gene expression data from the Cancer Cell Line Encyclopedia (CCLE; *n* = 883) and patient samples (*n* = 1643) from the INFORM registry study, we confirmed that these entities express low levels of *SLC7A11,* a previously described predictive biomarker for APR-246 responsiveness. Combining the CTRP drug response data with the respective CCLE gene expression profiles, we defined a novel gene signature, predicting the effectiveness of APR-246 treatment with a sensitivity of 90% and a specificity of 94%. We confirmed the predicted APR-246 sensitivity in 8/10 cell lines and in ex vivo cultures of patient samples. Moreover, the combination of ROS detoxification-impeding APR-246 with approved HDAC-inhibitors, known to elevate ROS, substantially increased APR-246 sensitivity in cell cultures and in vivo in two zebrafish neuroblastoma xenograft models. These data provide evidence that APR-246, in combination with HDAC-inhibitors, displays a novel potent targeted treatment option for neuroblastoma patients.

## 1. Introduction

Neuroblastoma is the most common extracranial solid tumor in childhood, accounting for 15% of pediatric cancer mortality [1]. Clinical characteristics of neuroblastoma cases are highly diverse concerning tumor size, progression, therapeutic response and prognosis. Treatment is implemented according to the respective risk group [2,3]. For low and intermediate risk tumors, which in general have a good prognosis, treatment ranges from observation and surgical resection to chemotherapy or radiation. Despite the intense therapeutic approach for high-risk neuroblastoma, the outcome is still poor, indicating a medical need for novel treatment options [4].

The tumor suppressor gene *TP53* is mutated in approximately 50% of all human cancers and to some extent nearly all cancers develop the ability to circumvent p53 pathway activation [5]. Primary neuroblastomas only rarely display *TP53* mutations, whereas relapsed high-risk neuroblastomas frequently show *TP53* mutations and loss of p53 function, which are both linked to a poor prognosis [6,7,8,9].

**Scheme 1:**
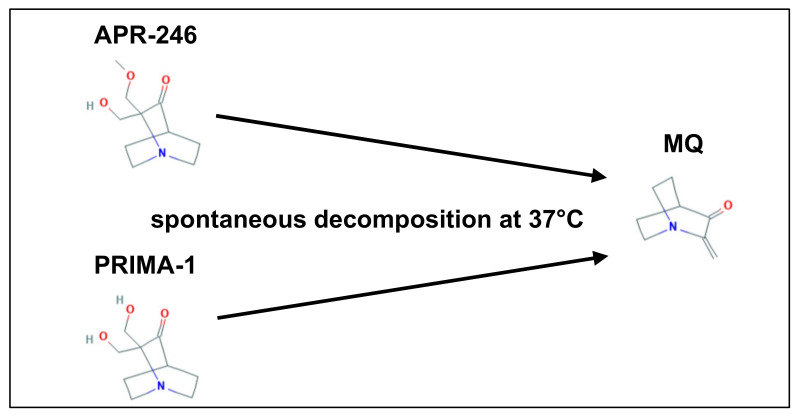
APR-246 and PRIMA-1 are prodrugs that are both spontaneously converted to highly reactive MQ in enzyme-free conditions [37,38,39].

Over the last 20 years, novel drugs were developed to reactivate mutated p53 [10]. The most advanced drug is APR-246, which is currently being tested in 12 clinical trials [11]. A phase III clinical study is assessing the efficacy of APR-246 in combination with azacytidine (NCT03745716). APR-246 is showing a very promising clinical potential, with a maximum plasma concentration of approximately 280 µM and a half-life of four to five hours, which corresponds to an estimated trough plasma concentration of 15–20 µM [12,13,14]. APR-246 is the methylated functional analogue of PRIMA-1, which was discovered in a cellular viability screening with an inducible expression system for mutant *TP53* in *TP53* knockout SAOS-2 cells [15]. Subsequent cell culture studies demonstrated that the prodrugs PRIMA-1 and APR-246 are converted spontaneously to reactive methylene quinuclidine (MQ) (Scheme 1) [16]. MQ covalently binds to cysteine residues of the mutant p53 (namely cysteine 124 and 277), which changes the protein’s conformation. This restores its DNA-binding function and induces a strong apoptotic signal [15,17].

In addition, MQ is able to bind other cysteine residues, e.g., thioredoxin reductase 1 (TrxR1) and glutathione [18,19]. MQ bound TrxR1 increases the ROS level via converting the enzyme to a pro-oxidant NADPH oxidase [18]. The tripeptide glutathione consists of glycine-cysteine-glutamate, of which cysteine is the active amino acid that is responsible for its antioxidant function. MQ binding impairs the antioxidant glutathione system and thus elevates the levels of reactive oxygen species (ROS) and lipid peroxidation, thereby promoting cell death [19]. Cysteine importers, such as SLC7A11 (also commonly known as xCT), are responsible for the intracellular amount of cysteine, and thus glutathione [19]. *SLC7A11* gene expression was identified as a response prediction biomarker for APR-246 treatment in esophagus carcinoma cell lines, whereby low expression levels of the cysteine importer are associated with a higher response to APR-246 treatment [19].

It is well-known that ROS levels can increase with cancer progression, for example through the induction of replicative stress [20]. In this case, elevated ROS levels can cause novel vulnerability in relapsed malignancies, i.e., in MAPK inhibitor resistant melanoma cells. In these cells, histone deacetylase (HDAC) inhibitors substantially elevated ROS levels and sensitized resistant cells for MAPK inhibitor treatment [21]. The enzyme family of HDACs is linked to oncogenic events and plays a major role in pediatric cancers of the nervous system [22,23,24,25,26]. Hence, HDAC inhibitors have demonstrated antitumor effects in various pediatric tumor models [27,28,29,30,31,32,33]. To date, several HDAC inhibitors are approved for the treatment of hematological cancers (e.g., vorinostat for cutaneous T-cell lymphoma and Panobinostat for multiple myeloma) and several clinical trials are investigating HDAC inhibitors as both single agents and in combination with chemotherapeutics, for additional tumor entities [34,35,36].

With this study, we aimed to investigate the effectiveness of APR-246 in the treatment of pediatric nervous system tumors in order to test and optimize predictive biomarkers and to show that ROS detoxification-impeding APR-246 is a promising potential combination partner for the ROS elevating HDAC inhibitors.

## 2. Materials and Methods

### 2.1. Cell Culture

Cell lines were cultured at 37 °C in a humidified atmosphere with 5% CO_2_. DNA fingerprinting authentication (DSMZ, Braunschweig, Germany) and bacterial and viral (including mycoplasma) contaminations were regularly checked (Multiplexion, Heidelberg, Germany). Adherent cell lines were detached using Trypsin (Gibco, Thermo Fisher Scientific Inc., Waltham, MA, USA).

The following neuroblastoma cell lines (all passage < 30) were cultured in Dulbecco’s Modified Eagle Medium (DMEM, Gibco, Thermo Fisher Scientific Inc., Waltham, MA, USA) with L-Glutamine supplemented with 10% fetal calf serum (FCS, Sigma-Aldrich, Munich, Germany) and 1% non-essential amino acids (NEAA, Lonza, Basel, Switzerland): SK-N-BE(2)-C (European Collection of Cell Cultures, ECACC), IMR-32 (German Collection of Microorganisms and Cell Cultures, DSMZ, Darmstadt, Germany), SK-N-AS (kindly provided by M. Schwab, DKFZ, Heidelberg, Germany), SH-SY5Y (DSMZ, Darmstadt, Germany). SK-N-FI (kindly provided by F. Westermann, DKFZ, Heidelberg, Germany), Kelly (DSMZ, Darmstadt, Germany)), IMR5/75 (kindly provided by F. Westermann, DKFZ, Heidelberg, Germany), SIMA (kindly provided by F. Westermann, DKFZ, Heidelberg, Germany), SH-EP (passage 15 to 30, kindly provided by F. Westermann, DKFZ, Heidelberg, Germany), SK-N-BE(2) (passage 15 to 30, DSMZ, Darmstadt, Germany) and NB-1 (passage 15 to 30, RIKEN Cell Bank, Japan) were cultured using RPMI 1640 with L-Glutamine (Gibco, Thermo Fisher Scientific Inc., Waltham, MA, USA), 10% FCS, 1% NEAA.

Vincristine resistant and non-resistant SK-N-BE(2)-C (passage 30 to 45, kindly provided by M. Michaelis, University of Kent, and J. Cinatl, Goethe University, Frankfurt am Main) were cultured using IMDM with L-Glutamine, 2 mM HEPES (Gibco, Thermo Fisher Scientific Inc., Waltham, MA, USA) supplemented with 10% FCS; for the resistant cells, 20 ng/mL vincristine was added to the medium. The cells were made to be resistant to vincristine-treatment through long-term cultivation in a vincristine containing medium [40].

The following pediatric high-grade glioma cell lines were used: SJ-GBM2 (passage 30 to 45, DMEM, 10% FCS, 1% NEAA), KNS-42 (passage 55 to 70, DMEM, 10% FCS, 1% NEAA), and SF188 (passage 95 to 110, DMEM, 10% FCS).

For the culture of Δ6RT 3T3 and C5-Gl 3T3 (passage 5 to 20 after obtaining, kindly provided by D. Pestov, Department of Cell Biology and Neuroscience, Rowan University School of Osteopathic Medicine, Stratford, NJ, USA), DMEM supplemented with 10% FCS was used [41].

The T-cell leukemia cell line Jurkat (passage 5 to 15 after obtaining, kindly provided by J. Hoheisel, DKFZ, Heidelberg, Germany) was cultured using RPMI 1640 with L-Glutamine (Gibco, Thermo Fisher Scientific Inc., Waltham, MA, USA), 10% FCS, 1% NEAA. The ALL cell lines NALM-6 (passage 5 to 15 after obtaining, kindly provided by T. Grünewald, DKFZ, Heidelberg, Germany) and REH (passage 5 to 15, kindly provided by T. Grünewald, DKFZ, Heidelberg, Germany) and the B-cell lymphoma cell line Jeko-1 (passage 5 to 15, kindly provided by M. Persicke, lab of D. Mertens, DKFZ, Heidelberg, Germany) were cultured using RPMI 1640 with L-Glutamine (Gibco, Thermo Fisher Scientific Inc., Waltham, MA, USA) supplemented with 10% FCS (Sigma-Aldrich, Munich, Germany). The T-cell lymphoma cell line HuT 78 was cultured with IMDM with L-Glutamine, and 2 mM HEPES (Gibco, Thermo Fisher Scientific Inc., Waltham, MA, USA) supplemented with 20% FCS.

Long-term cultures (LTC) were established from primary tumor samples obtained through the INFORM study (INdividualized Therapy FOr Relapsed Malignancies in Childhood [42]). The cells (passage 15 to 30 after isolation from the original tumor) were cultured using Tumor Stem Medium (TSM; [43]). The TSM consisted of filtered (Stericup-GP 500 mL Express Plus PES 0.22 µm, Merck, Darmstadt, Germany) TSM Base Medium (47.5% Neurobasal-A Medium, 47.5% D-MEM/F-12, 1% HEPES Buffer Solution (1 M), 1% sodium pyruvate MEM 100 MM(CE), 1% MEM Non-Essential Amino Acids Solution 10 mM, L-glutamine solution BIOXTRA, 2 mM, 1% Penicillin-Streptomycin-Glutamine, Life Technologies, Thermo Fisher Scientific Inc.) supplemented with 2% B27 Supplement Minus Vitamin A (50×, Life Technologies, Thermo Fisher Scientific Inc., Waltham, MA, USA), 0.02% (*w*/*v*) recombinant human EGF (100 µg/mL, PeproTech Inc., Rocky Hill, NJ, USA), 0.02% (*w*/*v*) recombinant human FGF-basic (100 µg/mL, PeproTech Inc., Rocky Hill, NJ, USA), 0.05% H-PDGF-AA (20 µg/mL, PeproTech Inc., Rocky Hill, NJ, USA) and 0.1% Heparin Solution (2 mg/mL, Sigma-Aldrich, Munich, Germany). The following cultures from INFORM patient samples were used: INF_R_1288_LTC, rhabdoid tumor (ATRT, MYC by methylation profiling); INF_R_1467_LTC, soft tissue sarcoma (eRMS by methylation profiling); INF_R_1490_LTC, osteosarcoma (OS_HG by methylation profiling) and INF_R_1632_PDX_LTC, neuroblastoma (NB, MYCN by methylation profiling). Ethics committee approval for INFORM was obtained from Heidelberg University Hospital’s review board.

Isolation and long-term cell culture establishment of PDX_LTC cells: INF_R_1632_PDX cells were isolated from a subcutaneous patient-derived xenograft (PDX) model established with a fresh surgical specimen of an INFORM patient with relapsed neuroblastoma according to a protocol adapted from Stewart et al. [44]. One tumor-bearing mouse was sacrificed when the volume of the subcutaneous tumor reached approximately 500 mm^3^. The harvested tumor tissue (260 mg) was minced thoroughly with sterile scissors, and was subsequently enzymatically digested for 10 min at 37 °C by incubating it with 1.2 µg/mL trypsin (Sigma-Aldrich, Munich, Germany) in Neurobasal-A medium (Life Technologies, Thermo Fisher Scientific Inc., Waltham, MA, USA). The reaction was stopped by adding 1.2 µg/mL trypsin inhibitor (Sigma-Aldrich, Munich, Germany), followed by the repeated stepwise addition of 60 µL 1 mg/mL DNAse in 0.5 M MgCl_2_ until viscosity of the solution was decreased, such that remaining tumor fragments easily settled to the bottom of the tube. After passing the cell suspension through a 40 µm cell strainer (Corning, Corning, NY, USA), the cells were spun down at 500 g for 10 min at room temperature. Red blood cells were removed by two cycles of 2 min incubation of the cell pellet in 2 mL ACK Lysing buffer (Lonza, Basel, Switzerland) at room temperature, followed by washing with a TSM base medium. The cells were taken into culture in TSM complete medium and formed free-floating three-dimensional spheroids and semi-adherent spheroids within 24 h after tumor dissociation. Free-floating and semi-adherent spheroids were dissociated with Trypl Express (Life Technologies, Thermo Fisher Scientific Inc., Waltham, MA, USA; 5 min, 37 °C) when they reached a size of 700–1000 µm, and the cells were sub-cultured at a ratio of 1:3 to 1:5 in fresh TSM complete. When the cells reached cell passage six, they were considered to be established and long-term cultures (INF_R_1632_PDX_LTC); we did not observe an obvious slowdown in cell growth over at least 35 passages in culture.

### 2.2. Chemicals

The following Table 1 summarizes the chemicals, reagents and diluents used.

### 2.3. Western Blot

Western blot analysis was performed as previously described [28]. The primary antibodies anti-p53 (sc-126, Santa Cruz Biotechnology, Dallas, TX, USA) and anti-GAPDH (JC1682928, Millipore, Darmstadt, Germany) were used. Peroxidase conjugated goat anti-mouse IgG (Fc specific) antibodies (A-0168, Sigma-Aldrich, Munich, Germany) were used as secondary antibodies. The original Western Blots data was shown in the Supplementary Materials.

### 2.4. Real-Time PCR

Real-time PCR was performed as described previously [45,46]. The following primers were purchased from ThermoFisher Scientific, Waltham, MA, USA:

*CDKN1A* (*p21^WAF1/CIP1^*, forward: 5′-TGG AGA CTC TCA GGG TCG AAA-3′, reverse: 5′-GGC GTT TGG AGT GGT AGA AAT C-3′), *HPRT* (forward: 5′-TGA CAC TGG CAA AAC AAT GCA-3′, reverse: 5′-GGT CCT TTT CAC CAG CAA GCT-3′), *SDHA* (forward: 5′-TGG GAA CAA GAG GGC ATC TG-3′, reverse: 5′-CCA CCA CTG CAT CAA ATT CAT G-3′), and *PUMA* (forward: 5′-CCT GGA GGG TCC TGT ACA ATC T-3′, reverse: 5′-GCA CCT AAT TGG GCT CCA TCT-3′). The primer for *GADD45A* was purchased from Qiagen, Hilden, Germany (QT00014084). The delta-delta-Ct method was used to calculate the relative expression, and normalization was performed to neuroblastoma specific housekeeping genes *SDHA* and *HPRT* [45,47].

### 2.5. Flow Cytometry Analysis

Flow Cytometry was applied to analyze the lipid peroxidation (BODIPY staining) and to detect ROS (DCFDA staining).

BODIPY 581/591 C11 Lipid peroxidation sensor staining (stock concentration: 20 mM in dimethylformamide, Invitrogen, Thermo Fisher Scientific, Waltham, MA, USA) was used at a working concentration of 20 µM, and was diluted with RPMI without phenol red (Gibco, Thermo Fisher Scientific Inc., Waltham, MA, USA). After inspection, the cells were detached, centrifuged and stained for 20 min at 37 °C in a 500 µL staining solution. After centrifugation, cells were resuspended in 300 µL RPMI without phenol red, supplemented with 10% FCS. Fluorescence was measured in a FACSCanto II (BD) with 488 nm excitation and detected using a 502 nm longpass and 530/30 nm bandpass filter. Data were analyzed using FlowJo 10.x (FlowJo LLC, Ashland, OR, USA).

ROS detection was performed as previously described [27].

### 2.6. Metabolic Activity Assays

If not otherwise declared, cells were precultured for 72 h at a density of 2,000,000 cells per T75 flask (exceptions: Kelly, NB1 and IMR-32 4,000,000 per T75 flask, SJ-GBM2 1,000,000 per T75 flask). Cells were detached with trypsin—EDTA (Thermo Fisher Scientific, Waltham, MA, USA) and seeded in 96-well plates (Greiner, Microplate, 96-Well, PS, F-bottom µCLEAR^®^, CELLSTAR^®^) in 100 µL medium per well at a density of 5000 (KNS-42, SJ-GBM2, SF188, SK-N-BE(2)-C) or 10,000 (IMR-32, NB1, Kelly) cells/well. HDAC-inhibitor screenings were performed accordingly in 384-well plates (Greiner, Microplate, 384-Well, PS, F-bottom µCLEAR^®^, CELLSTAR^®^) in 20 µL medium per well at a density of 1250 (SK-N-BE(2)-C, SK-N-AS) or 2500 (IMR-32) cells/well. To avoid edge effects, margin wells (row A and H, column 1 and 12) contained PBS or medium and medium-containing wells were used as background controls. After 24 h incubation, cells were treated for 72 h. Drugs were applied using a Tecan D300e drug printer. Cell viability was quantified using CellTiter-Glo^®^ 2.0 kit (Promega, Madison, WI, USA). 50 µL CellTiter-Glo^®^ (96-well) or 25 µL CellTiter-Glo^®^ (384-well) were added to every well, the plates were shaken at 400 rpm for 5 min and were then incubated for another 10 min. Bioluminescence was quantified on an OPTIMA plate reader (BMG Labtech, Ortenberg, Germany).

### 2.7. Trypan Blue Assay

An automated Trypan Blue Assay was performed as previously described [30].

### 2.8. Zebrafish Lines

The care for and breeding of the zebrafish were done under standardized conditions, as described previously [48]. Zebrafish wild-type AB line was raised at 28 °C. Embryos used for tumor injections were maintained in an E3 buffer supplemented with 0.2 mM 1-phenyl-2- thiourea (PTU, Sigma, Munich, Germany).

### 2.9. Cell Preparation for Zebrafish Embryo Xenotransplantation

Cell preparation and xenotransplantation were performed as described previously [48]. Briefly, cells (NB-1 and IMR-32) were cultured to 70–80% confluence, and then harvested and labeled with CellTracker CM-DiI (Thermo Fisher Scientific, Waltham, MA, USA). To minimize cell clumping, DNase I (250 Kunitz units/mL, Sigma-Aldrich, Munich, Germany) for IMR-32 and Benzonase (E1014-25KU, Sigma-Aldrich, Munich, Germany) for NB-1 was added to the cell suspension and washed twice with 10% FCS RPMI, twice with serum-free RPMI and resuspended in serum-free RPMI to a final concentration of 1.0 × 10^8^ cell/mL. Zebrafish embryos were anesthetized with tricaine (MS-222, 3-Amino-benzoesäure-ethylester-methansulfonat, 0.02% (*w*/*v*), Sigma-Aldrich, Munich, Germany) and embedded in 1.0% of low gelling temperature agarose (Sigma-Aldrich, Munich, Germany). 150 to 250 CM-DiI-labeled tumor cells were injected into the yolk sac of each embryo using a FemtoJet express microinjector (Eppendorf, Hamburg, Germany) and glass microinjection needles (Science Products, Hofheim, Germany). Shortly (30 min) after injection, embryos were transferred to and held at 34 °C.

### 2.10. Zebrafish Embryo Drug Toxicity Assay

The toxicity of the compounds was assessed before performing in vivo experiments and a Maximum Tolerated Dose (MTD) was determined. Embryos (48 hpf) were transferred to 48-well uncoated plates (Corning, Corning, NY, USA) containing different concentrations of the compounds and a solvent control. The stock solution of the compounds was further dissolved in E3 buffer supplemented with 0.2 mM 1-phenyl-2-thiourea (PTU, Sigma, Munich, Germany) in order to reach the desired concentrations. Three embryos were tested per concentration or solvent control. Embryos were kept at 34 °C during the experiment and were imaged at 72 hpf and 120 hpf using a stereo microscope (Leica). The embryos were assessed for signs of toxicity, such as death, morphology changes (curvature of body, edema) and behavioral changes. APR-246 concentration range tested: 10–200 µM, MTD: 50 µM; solvent control: DMSO (0.2% and 0.5%); Vorinostat concentration range tested: 2.5–100 µM, no toxicity detected (MTD: 100 µM); solvent control: DMSO (0.1%).

### 2.11. Zebrafish Embryo Drug Treatment and Efficiency Evaluation

Drug treatment and efficiency evaluation were performed as described previously [48]. Briefly, embryos with tumor xenografts, detected by red signals in fluorescence microscopy (Nikon, Tokyo, Japan) 2 h post implantation, were transferred to 48-well uncoated plates (Corning, Corning, NY, USA). Then, embryos were incubated in freshly prepared E3 medium supplemented with 1% N-Phenylthiourea (PTU, Sigma-Aldrich, Munich, Germany) containing drugs or solvent control. Tumor growth was evaluated by confocal microscopy before drug exposure and 48 h post treatment (Zeiss LSM 710 confocal microscope (Zeiss, Oberkochen, Germany) and ZEN software (Zeiss, Oberkochen, Germany)). The responses of tumor volumes were categorized according to the Response Evaluation Criteria in Solid Tumors (RECIST) 1.1, which was adopted for zebrafish tumors, to visualize best response: progressive disease (PD, at least a 20% increase in tumor volume), stable disease (SD, neither sufficient shrinkage to qualify for PR nor sufficient increase to qualify for PD), and partial response (PR, at least a 30% decrease in tumor volume).

### 2.12. In Silico Data Analysis and Genetic Signature Generation

We downloaded drug response data for *n* = 1107 cell lines to PRIMA-1 from the Cancer therapeutics response CTD^2^ portal (Broad Institute, Cambridge, MA, USA, CTRP v2, 2015) [49,50,51,52]. The data were combined with gene expression and *TP53* mutation data for the respective cell lines and entities from the Cancer Cell Line Encyclopedia (CCLE, Broad Institute, Cambridge, MA, USA, *n* = 883 from 1375 CCLE cell lines had TP53 data on the CTRP); doublets were excluded. We first used drug response data to discriminate between the cell lines that were responsive (area under curve below 11, reflecting an app. IC50 of less than 10 µM, *n* = 102 cell lines) and less-responsive (area under curve above 13, reflecting an app. IC50 of more than 40 µM, *n* = 303 cell lines) to PRIMA-1 treatment. We then looked for differentially expressed genes in the responsive versus less-responsive cohorts using the Cancer Cell Line Encyclopedia (CCLE, Broad Institute, Cambridge, MA, USA) gene expression data set in R2 (R2: microarray and visualization platform: http://r2.amc.nl, accessed on 5 January 2021).

Using z-scores of log2 transformed gene expression data, we identified *n* = 171 genes as the most significantly differentially expressed genes between the two cohorts (*p*-value < 2.82 × 10^−31^, *n* = 16 upregulated and *n* = 155 downregulated genes). We tested every gene on this list for its suitability as a single gene predictive biomarker against the whole CCLE cohort. Downregulated genes were characterized by a negative overall fold change; upregulated genes were characterized by a positive overall fold change. Whether a gene in a respective cell line was up- or downregulated was reflected by its z-score. Based on how often these predictions were true, we calculated the sensitivity (true positive prediction rate), specificity (true negative prediction rate) and general false prediction rate using an R script (Supplementary Materials).

To investigate the predictive power of the combinations of several genes, we first analyzed the up- and downregulated genes separately:

For upregulated genes (*n* = 16), we tested every possible combination of genes by calculating the sum of the z-scores for every cell line. We assumed that positive and negative sums of z-scores would predict the responders and low-responders, respectively. We then calculated the sensitivity, specificity and false prediction rate by comparing the number of predicted responders and low-responders to the actual responder vs. low-responder status. The best combination, with 84.3% sensitivity, 96.7% specificity and a 5.0% false prediction rate, was then used for the ratio calculation described below.

Due to the high number of downregulated genes (*n* = 155), we analyzed a limited number of 40 downregulated genes: 20 with the lowest false prediction rate and 20 with the highest sensitivity from the single gene analysis. These 40 genes were combined in every possible way up to a group size of five. Sensitivity and specificity of every combination were calculated under the following assumptions: A negative sum of z-scores predicted responders and a positive sum of z-scores predicted low-responders. Predictions and true responder/low-responder status were compared. Based on the number of correctly predicted responder and low-responder sensitivity, specificity and false prediction rate were calculated. The best combination, with 90.2% sensitivity, 94.7% specificity and a 5.0% false prediction rate, was then used for the ratio calculation.

To merge the best above identified combinations of down- and upregulated genes, we divided the sum of the upregulated gene expression values (log2 transformed) by the sum of downregulated gene expression values (log2 transformed). To identify a cut-off value for the discrimination of responders from low-responders, we used the receiver operating curve (ROC) analysis (ROCR R-package). Samples with a ratio > ROC cut-off are predicted as responders, samples with a ratio < cut-off are predicted as low-responders. The R-package compared the prediction to real response from the Cancer Therapeutics Response Portal and calculated the sensitivity, specificity and false prediction rate.

By using the ratio of log2 transformed gene expression values of the respective genes, it was possible to calculate a genetic signature score (“biomarker”) for any cohort of interest (e.g., INFORM).

### 2.13. Stastical Analyses

All cell culture experiments were performed in triplicate. A two-tailed *t*-test, unpaired or paired, or ANOVA where appropriate, were performed using software program R (R version 3.2.2, 2015; The R Foundation for Statistical Computing). The packages tidyverse, janitor, readxl, writxl, ROCR, ggplot2 and ggsignif were used to calculate and plot the data. *p*-values of less than 0.05 were considered significant (* *p* < 0.05, ** *p* < 0.01, *** *p* < 0.001). IC50 values were calculated using GraphPad Prism Version 9.1.0 (GraphPad Software, San Diego, CA, USA). Synergistic interactions were analyzed with SynergyFinder using the HSA (highest single agent) reference model, as the assumptions of other models were not fulfilled [53].

## 3. Results

### 3.1. Pure TP53 Mutation Status Is Not a Sufficient Biomarker for APR-246 Sensitivity of Pediatric Tumors of the Nervous System

**Figure 1:**
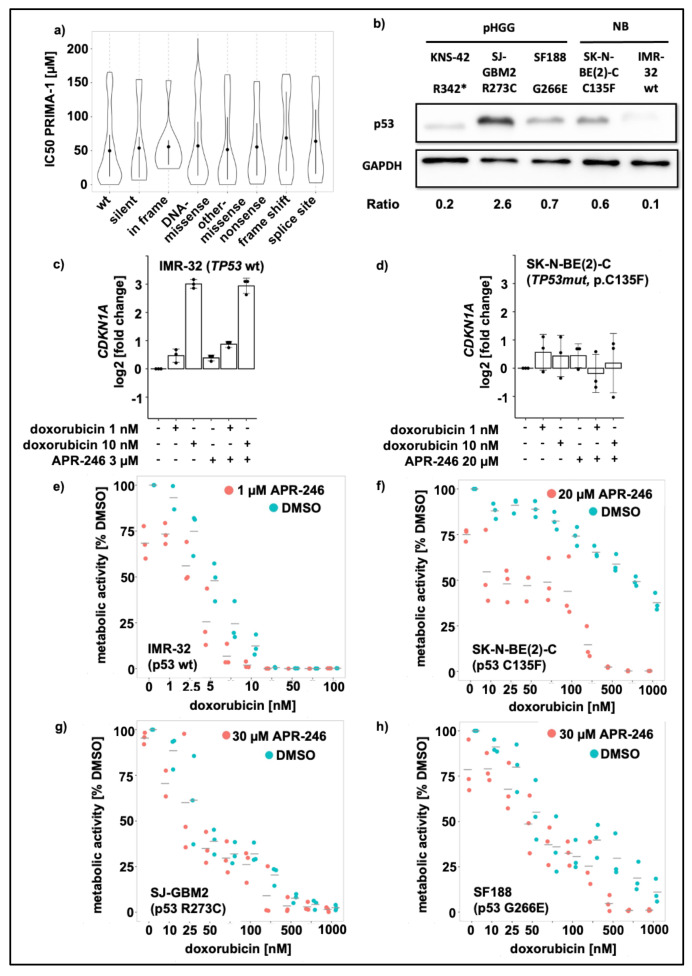
*TP53* mutation status insufficiently predicts the APR-246/PRIMA-1 response. (**a**): PRIMA-1 sensitivity separated according to *TP53* mutation status derived from Cancer Cell Line Encyclopedia and CTD^2^ portal (*n* = 805); (**b**): Expression of p53 is shown in the Western blot of the whole cell lysates of one *TP53* nonsense mutated cell line (KNS-42), three *TP53* missense mutated cell lines (SJ-GBM2, SF188, SK-N-BE(2)-C) and one *TP53* wild type cell line (IMR-32). GAPDH served as loading control. The ratio indicates normalization of p53 to the GAPDH signal; (**c**,**d**): RT-PCR measurement of *TP53* pathway activation. *CDKN1A* expression is depicted as log2 transformed fold change to solvent control. Treatment was applied for 24 h with doxorubicin or APR-246, alone or in combination, as indicated; (**e**,**f**): metabolic activity measurement with CellTiter Glo-assay in neuroblastoma cell lines IMR-32 (**e**) and SK-N-BE(2)-C (**f**) after 72 h treatment. IMR-32 (**e**) were treated with 0–100 nM doxorubicin alone (blue) or in combination with a fixed APR-246 concentration of 1 µM (red). SK-N-BE(2)-C (**f**) were treated with 0–1000 nM doxorubicin alone (blue) or in combination with a fixed APR-246 concentration of 20 µM (red). Luminescence was normalized to the solvent control (DMSO). (**g**,**h**): metabolic activity measurement with CellTiter Glo-assay in pediatric high-grade glioma cell lines SJ-GBM2 (**g**) and SF188 (**h**) upon 72 h treatment. Both were treated with 0–1000 nM doxorubicin alone (blue) or in combination with a fixed APR-246 concentration of 30 µM (red). Luminescence was normalized to the solvent control (DMSO).

While developed as a p53 stabilizing agent, it has emerged that *TP53*’s mutation status alone is not a satisfactory biomarker for APR-246/PRIMA-1 sensitivity, e.g., in Ewing sarcoma and esophagus carcinoma models [54,55]. To investigate APR-246/PRIMA-1 responsiveness and the predictive value of the *TP53* mutation status for childhood tumors, we applied in silico data analysis. We combined the data for the *TP53* mutation status of a large cohort of cancer cell lines (including pediatric entities such as medulloblastoma, neuroblastoma and Ewing sarcoma) from the Cancer Cell Line Encyclopedia (CCLE, Broad Institute, Cambridge, MA, USA) and the DepMap portal with PRIMA-1 sensitivity data obtained from the CTD^2^ portal (Broad Institute, Cambridge, MA, USA, CTRP v2, 2015) [49,56,57]. If the *TP53* mutation type was the main driver of responsiveness, sensitivity to PRIMA-1/APR-246 would be expected in cell lines that harbor *TP53* point mutations leading to a conformational change impairing DNA binding, as this can be reversed by APR-246/PRIMA-1 [14,15,16]. These include the missense mutations R273H, R273C, C135F and G266E [58]. In line with the studies mentioned above, our analysis revealed no increased sensitivity in cell lines (total as well as pediatric-only) harboring these missense mutations (Figure 1a, Figure S1).

Moreover, the wet-lab results from five pediatric tumor cell lines treated with APR-246 proved no correlation of IC50 values with the *TP53* mutation status. In fact, the *TP53* wild type cell line (IMR-32) was the most sensitive of all tested cell lines, as determined by metabolic activity readout (Table 2). We used three cell lines that harbor common *TP53* missense mutations within their DNA-binding domain (R273C, G266E and C135F), one cell line with a nonsense mutation (R342*, DNA-binding function remains intact), and one *TP53* wild-type cell line (Table 2). The Western blot analysis revealed the accumulation of p53 protein, which is typical for *TP53* mutation, for SJ-GBM2 (R273C), SF188 (G266E) and SK-N-BE(2)-C (C135F) cells (Figure 1b). To further investigate the contribution of p53 for APR-246 sensitivity, we treated NIH3T3-*TP53* knockout cells and the corresponding NIH3T3-*TP53* wild-type cells with APR-246. Both cell types equally responded to APR-246 treatment, independently of the *TP53* status (Table 2).

To determine whether APR-246 exerts its p53-activating potential, we combined APR-246 with doxorubicin, which is a well-known DNA damage and p53 response inducer. Doxorubicin single-treatment activated p53 in the *TP53*-wt cell line IMR-32, as indicated by the upregulation of the p53 target genes *CDKN1A, PUMA* and *GADD45A* (Figure 1c; Figure S2). The addition of APR-246 to doxorubicin treatment did not increase target gene expression in the *TP53*-wildtype setting when compared to cells treated with doxorubicin only (Figure 1c; Figure S2). In the *TP53* mutant neuroblastoma cell line SK-N-BE(2)-C (C135F), doxorubicin single treatment failed to activate p53 target gene expression. Furthermore, the addition of APR-246 to doxorubicin did not improve p53 target gene induction in this cell line (Figure 1d; Figure S2). Nevertheless, when combining a fixed APR-246 concentration with different doses of doxorubicin, we observed increased doxorubicin sensitivity in both the *TP53*-wt IMR-32 cell line (Figure 1e) and in the doxorubicin resistant *TP53*-mutant SK-B-BE(2)-C cell line (Figure 1f). Our metabolic activity data thus suggest that the combination of APR-246 with doxorubicin reduces viability in both *TP53*-wt and mutant neuroblastoma cell lines, despite the absence of a p53 target gene expression response in the *TP53*-mutant cell line.

These findings were confirmed in *TP53* mutant pHGG cell lines SJ-GBM2 and SF188 (Figure 1g–h). Similar to SK-N-BE(2)-C cells, both cell lines exhibited decreased cell viability upon doxorubicin treatment combined with APR-246. Again, we observed no reactivation of p53 target gene expression (*CDKN1A* and *GADD45A*) upon APR-246 treatment in these cells (Figure S3). Altogether, these results suggest that the *TP53* mutation status alone is not sufficient to predict the response to APR-246 alone or in combination with doxorubicin in childhood tumors of the nervous system.

### 3.2. APR-246 Impairs Oxygene Species Elimination and Basal Reactive Oxygen Species Level Indicate APR-246 Sensitivity

**Figure 2:**
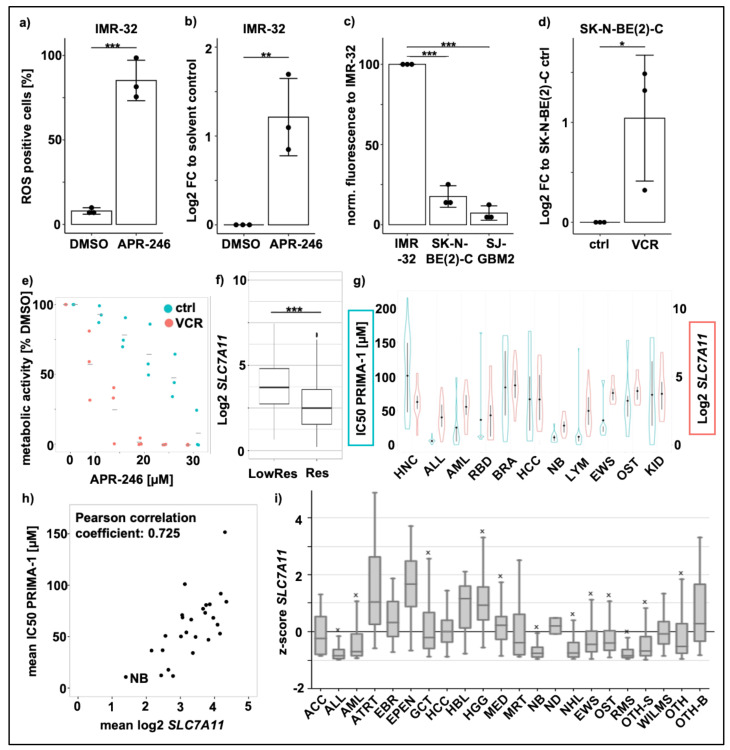
APR-246 response depends on the ROS levels. (**a**): The percentage of ROS positive IMR-32 cells after treatment with 50 µM APR-246 for 5 h compared to solvent control (DMSO) detected after DCFDA staining by FACS; (**b**): Log2 fold change of BODIPY positive IMR-32 cells after treatment with 50 µM APR-246 for 5 h compared to solvent control (DMSO) detected by FACS; (**c**): Basal ROS level in IMR-32, SK-N-BE(2)-C and SJ-GBM2 detected after DCFDA staining by FACS. Data were normalized to IMR-32 signal; (**d**): Log2 fold change of basal ROS level in vincristine resistant SK-N-BE(2)-C compared to control SK-N-BE(2)-C detected after DCFDA staining by FACS; (**e**): Metabolic activity measurement with CellTiter Glo-assay in vincristine resistant SK-N-BE(2)-C (red) and control SK-N-BE(2)-C (blue) after 72 h treatment with APR-246 0–30 µM. Luminescence was normalized to the respective solvent control (DMSO); (**f**): Log2 *SLC7A11* expression of PRIMA-1 less responsive cell lines (LowRes, IC50 < 20 µM) compared to responsive cell lines (Res, IC50 > 20 µM) from the Cancer Cell Line Encyclopedia (*n* = 744); (**g**): Combination of log2 *SLC7A11* expression and PRIMA-1 sensitivity stratified by cancer cell line entities with data obtained from Cancer Cell Line Encyclopedia and CTD^2^ portal (*n* = 229); (**h**): Plot of the mean log2 SLC7A11 expression of entities (*n* = 26) from the CCLE against the mean PRIMA-1 IC50 of the respective entities. Pearson correlation coefficient was calculated using R software; (**i**) Z-scores of *SLC7A11* log2 expression from INFORM patient samples stratified by entities and analyzed with R2 (*n* = 1643). ACC: adrenocortical carcinoma, ALL: acute lymphoblastic leukemia, AML: acute myeloid leukemia, ATRT: atypical teratoid/rhabdoid tumor, BRA: Brain cancer, EBR: embryonal brain tumor, EPEN: ependymoma, GCT: germ cell tumor, HCC: hepatocellular carcinoma, HBL: hepatoblastoma, HCC: Liver cancer, HGG: high-grade glioma, HNC: Head and Neck Cancer, KID: Kidney Cancer, LYM: Lymphoma, MED: medulloblastoma, MRT: malignant rhabdoid tumor, NB: neuroblastoma, ND: not defined, NHL: non-Hodgkin lymphoma, EWS: Ewing sarcoma, OST: osteosarcoma, RBD: Rhabdoid, RMS: rhabdomyosarcoma, OTHS: other sarcoma, WILMS: nephroblastoma, OTH: other, OTHB: other brain tumor. * *p* < 0.05, ** *p* < 0.01, *** *p* < 0.001.

Besides binding to mutant p53, the APR-246/PRIMA-1 product MQ has been described to covalently bind to and inactivate the antioxidant tripeptide glutathione [19]. Additionally, MQ has been shown to inhibit thioredoxin reductase 1 (TrxR1) resulting in a pro-oxidant NADPH-oxidase [18]. These effects result in increased levels of reactive oxygen species (ROS) [18,19]. We observed increased ROS formation and lipid peroxidation in our neuroblastoma models upon APR-246 treatment (Figure 2a,b). In addition, we compared basal ROS levels of responsive IMR-32 as well as less responsive SK-N-BE(2)-C and SJ-GBM2 cells. Whereas the sensitive IMR-32 cells displayed very high basal ROS levels, the less-responsive cell lines exhibited only low basal ROS levels, suggesting that cells with a high degree of basal oxidative stress respond better to the treatment (Figure 2c). To test this assumption, we analyzed a vincristine resistant and vincristine non-resistant neuroblastoma cell line pair, generated through long-term cultivation of the parental cell line in vincristine or solvent containing medium [40]. Consistent with our hypothesis, the chemotherapy resistant cell model displayed significantly increased ROS-levels, and in turn increased APR-246 sensitivity, when compared to its chemotherapy non-resistant control counterpart (Figure 2d,e).

ROS levels are controlled by intracellular glutathione levels and cysteine importers, such as the cystine-glutamate antiporter encoded by the *SLC7A11* gene. Consequently, low *SLC7A11* expression levels correlate with low amounts of glutathione and high ROS levels. Low amounts of glutathione are completely bound by APR-246/MQ and the expression of *SLC7A11* has been proposed as a predictive biomarker of APR-246 responsiveness in esophagus carcinoma cell lines [19].

To test *SLC7A11* as a response prediction marker for APR-246 sensitivity in pediatric nervous system tumors, we first performed an in silico data analysis of responsive and less-responsive cell lines of the whole Cancer Cell Line Encyclopedia (CCLE), including all cell lines. We divided the cell lines into two groups according to their sensitivity. Based on pharmacokinetics data, we defined the maximum APR-246 trough plasma level of 20 µM in order to discriminate between responders and low-responders [12]. We then analyzed the *SLC7A11* gene expression in the two CCLE groups [56,57]. Indeed, *SLC7A11* expression was significantly lower in responsive versus low-responsive cell lines (Figure 2f).

In a reverse approach, we investigated which cell lines from which entities displayed low *SLC7A11* expressions. In particular, cell lines of the pediatric entities neuroblastoma and acute lymphoblastic leukemia, as well as adult entities lymphoma and acute myeloid leukemia, expressed *SLC7A11* at a low level. We hypothesized that the low *SLC7A11* expression was associated with a higher responsiveness of these entities to APR-246/PRIMA-1. Combining the expression and sensitivity data confirmed this assumption (Figure 2g, Figure S5). Moreover, *SLC7A11* expression levels of each entity correlated well with their mean APR-246 sensitivity (Pearson correlation coefficient of 0.725) (Figure 2h). Taken together, a low *SLC7A11* expression indicates a high APR-246/PRIMA-1 responsiveness.

Analyzing expression data from the Individualized Therapy For Relapsed Malignancies in Childhood (INFORM) register study using the R2 bioinformatic data analysis platform, we could confirm that neuroblastoma patient samples sensitive to APR-246/PRIMA-1 are characterized by very low *SLC7A11* levels, as compared to most other pediatric entities. Notably, acute lymphoblastic lymphoma (ALL) and non-Hodgkin lymphoma (NHL) showed a similarly low expression (Figure 2i) [42].

To verify our in silico analysis with wet-lab data, we tested seven randomly picked cell line models (four neuroblastoma, two ALL and one non-Hodgkin lymphoma (NHL) cell lines) for APR-246 sensitivity. Six of the seven models were highly responsive to treatment with APR-246 (Table 3).

Taken together our data show that APR-246 treatment results in oxidative stress and cell death and that oxidative stress sensitizes cells to APR-246 treatment. A low *SLC7A11* expression indicates a high APR-246 responsiveness. Entities with rather low *SLC7A11* expression levels (CCLE data) are neuroblastoma, ALL or NHL. A similar *SLC7A11* expression pattern was observed in the INFORM patient cohort, with ALL, rhabdomyosarcoma, NHL and neuroblastoma being those with the lowest SLC7A11 expressions.

### 3.3. A Novel Biomarker Signature Improves Response Predicition for Neuroblastoma Cell Lines and Patients

**Figure 3:**
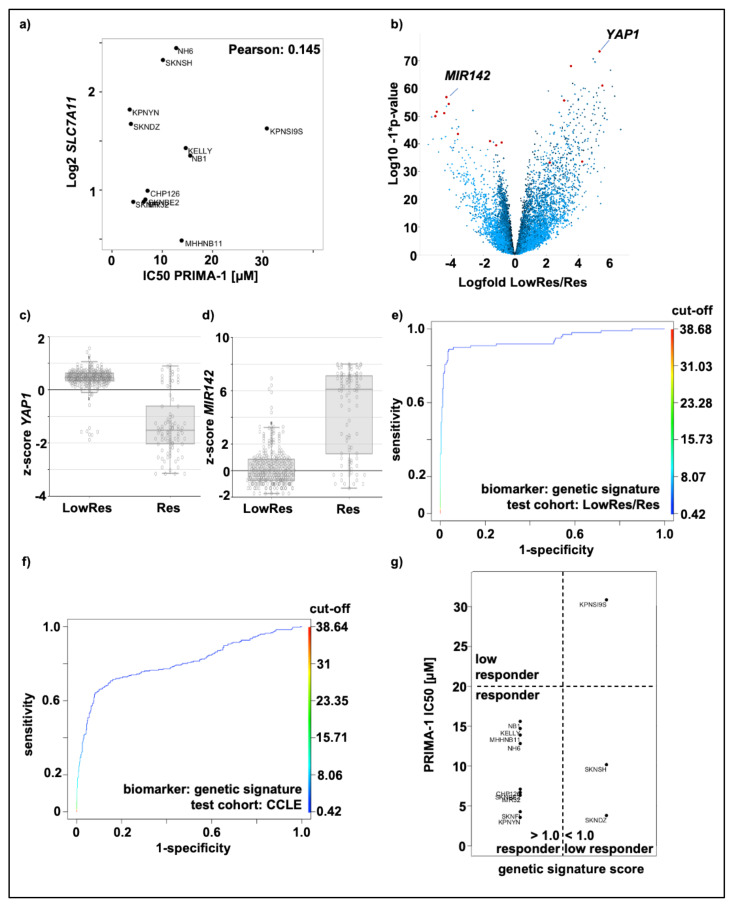
Identification of a biomarker predicting APR-246 responsiveness (**a**): in silico analysis of log2 *SLC7A11* and PRIMA-1 sensitivity (IC50 in µM) with data obtained from Cancer Cell Line Encyclopedia and CTD^2^ portal for available neuroblastoma cell lines. The Pearson correlation coefficient (0.145) was calculated using R software (*n* = 12); (**b**): volcano plot of differentially expressed genes between less responsive cell lines (PRIMA-1 IC50 app. > 40 µM, *n* = 303) and responsive cell lines (PRIMA-1 IC50 app. < 10 µM, *n* = 102) calculated using R2 software. Except for *YAP1* and *MIR142* (most significantly down and up-regulated gene respectively) all red dots indicate genes that are part of the genetic signature; (**c**,**d**): boxplot of z-score of in responsive cell lines that were the most significantly downregulated (**c**, *YAP1*) and upregulated (**d**, *MIR142*) genes plotted with R2; (**e**,**f**): receiver operating characteristic (ROC) curve calculated with ROCR package in R software for the genetic signature score to discriminate between low-responders (PRIMA-1 IC50 app. > 40 µM, *n* = 303) and responders (PRIMA-1 IC50 app. < 10 µM, *n* = 102) (**e**) or to discriminate the IC50s less than or greater than 20 µM (**f**). Responder and low-responder status (*n* = 405) (**e**) or the whole CCLE cell lines (*n* = 711) with known PRIMA-1 sensitivity data were used as test cohort (**f**); (**g**): in silico analysis of genetic signature score predicting PRIMA-1 responsiveness in neuroblastoma sub-cohort from CCLE (*n* = 12). PRIMA-1 sensitivity cut-off 20 µM was chosen. Ten out of twelve cell lines were predicted correctly. SK-N-SH and SK-N-DZ were falsely predicted to be low-responders. LowRes: low-responder (*n* = 303), Res: responder (*n* = 102).

While *SLC7A11* indicates responsiveness quite well for tumor entities, we noticed that the correlation between *SLC7A11* expression and APR-246/PRIMA-1 sensitivity (IC50) was reduced in neuroblastoma cell lines (Pearson correlation coefficient of 0.145, Figure 3a).

To screen for additional novel APR-246/PRIMA-1 response prediction biomarkers in neuroblastoma, we defined two response groups based on the 20 µM plasma trough level threshold: high responders (app. IC50 < 10 µM, 50% plasma trough level) and low-responders (app. IC50 > 40 µM, 200% plasma trough level). We screened R2 gene expression data sets (CCLE) for genes that are differently expressed between high and low-responders (Figure 3b). Among the 171 most significantly differentially expressed genes, 155 genes showed a negative fold change from low-responders to high responders, meaning that these genes were downregulated in responders; 16 genes showing a positive fold change were accordingly upregulated in responders.

The top downregulated gene indicating APR-246 sensitivity was *YAP1* (*p*-value 1.08 × 10^−69^, fold change −2.32, Figure 3c). The top upregulated gene indicating sensitivity was *MIR142* (*p*-value 1.28 × 10^−58^, fold change 17.75, Figure 3d). We tested the predictive value of the identified genes to classify high-responder/low-responder, resulting in 79.4% (*YAP1*) and 73.5 % (*MIR142*) sensitivity and 84.8% (*YAP1*) and 86.1% (*MIR142*) specificity. To generate a biomarker with improved sensitivity and specificity, we combined highly differentially expressed genes to the following gene signature and calculated a signature score according to this formula (sum of upregulated genes divided by sum of downregulated genes):

(log2*CORO1A* + log2*RHOH* + log2*CXCR4* + log2*PUM2* + log2*TRAF3IP3* + log2*KDM2B* + log2*PDE7A* + log*2ARHGAP9*)/

(log2*PLS3* + log2*RHBDF1* + log2*CLDN1* + log2*SLC7A11* + log2*FAM114A1*)

The optimal cut-off value for a biomarker discriminating between a high responder and low-responder was 1.1, as determined with a receiver operating characteristic (ROC) curve. Higher values indicate higher responsive samples and lower values indicate less responsive samples. The sensitivity at this cut-off was 90.0% and the specificity was 94.3% (Figure 3e). In the next step, we applied the identified gene signature to all cell lines in the CCLE with available sensitivity data and chose the maximum APR-246 plasma trough level of 20 µM as the sensitivity threshold to be predicted [12]. With this threshold, the most efficient biomarker cut-off was determined to be 1.0, giving a sensitivity of 71.5% and a specificity of 83.8% (Figure 3f). With this biomarker at hand, we again investigated the neuroblastoma sub-cohort of the CCLE and found that 10 out of the 12 models were correctly predicted (Figure 3g).

We further challenged the biomarker’s predictions and acquired our own cell culture data from three neuroblastoma models with known expression data, but with unknown sensitivity: SIMA (biomarker 1.80), SH-SY5Y (biomarker 1.68) and SK-N-AS (biomarker 1.03). We expected a cell line with a signature score higher than 1.0 to have an IC50 of below 20 µM and vice versa. The criteria were met in two of three neuroblastoma cell lines. We also tested three more models of other entities as well as four cultures derived from INFORM patient samples. Notably, the neuroblastoma long-term culture was derived from a neuroblastoma PDX model of an INFORM patient. We identified the APR-246 sensitivity and compared it to the calculated genetic signature score. All three established cell lines (KNS-42, Jurkat, JeKo-1) met the criteria, and three of the four INFORM samples were correctly predicted (Table 4).

Notably, only 35.5% of all cell lines in the CCLE (*n* = 1375) were predicted to be responders according to our signature score. In contrast, 90.0% of CCLE neuroblastoma cell lines were predicted to be responsive, again highlighting the pronounced sensitivity of this entity to APR-246 treatment.

### 3.4. Sensitizing Low-Responsive Neuroblastoma Cells to APR-246 Using HDAC-Inhibitors

**Figure 4:**
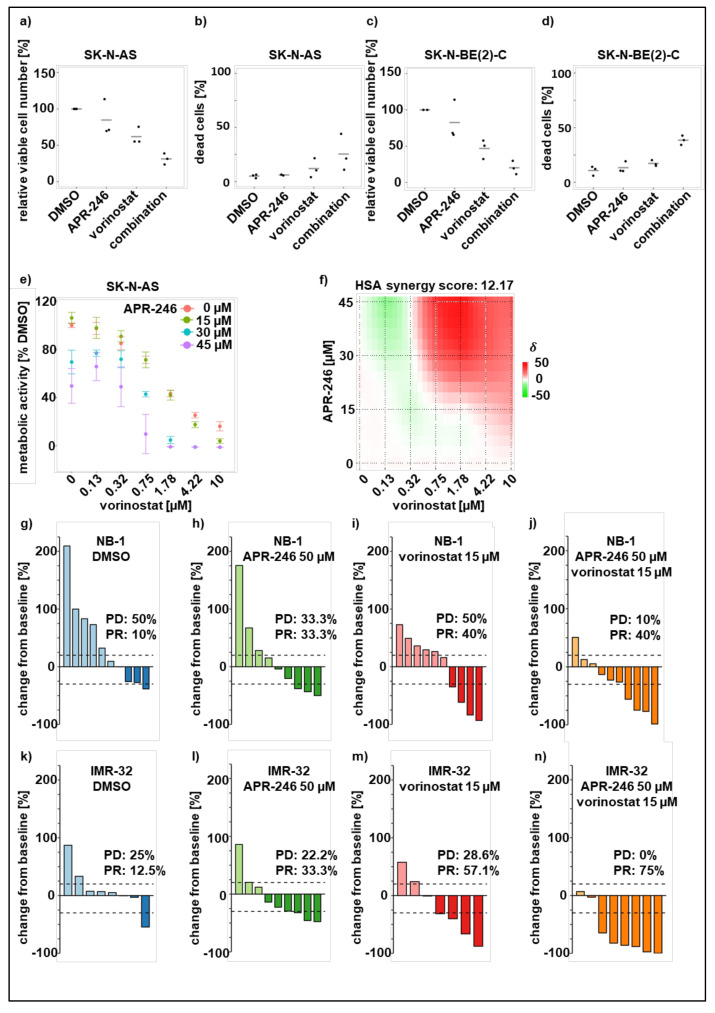
Combining APR-246 with HDAC inhibition effectively sensitizes neuroblastoma in vitro and in vivo. Determination of viable cell number/mL normalized to solvent (DMSO)-treated cells (**a**,**c**) and dead cell count (**b**,**d**) after automated Trypan blue exclusion assay of neuroblastoma cell lines SK-N-AS and SK-N-BE(2)-C. Cells were pretreated with 0.5 µM vorinostat for 72 h and afterwards treated with 20 µM APR-246 for 24 h. Bars indicate control, single and sequential treatment; (**e**,**f**): metabolic activity measurement via CellTiter Glo-assay in neuroblastoma cell line SK-N-AS after 72 h treatment with 0–10 µM vorinostat combined with fixed APR-246 concentrations of 0 µM, 15 µM, 30 µM and 45 µM. Normalization was performed to the solvent control (DMSO). Synergy calculation (**f**) was performed with synergyfinder (https://synergyfinder.fimm.fi, accessed 30 April 2020) by applying HSA (highest single agent) model; (**g**–**n**): waterfall plots demonstrating the change in tumor volume (%) for each individual xenograft, from baseline (day 1 = start of the treatment) to day 3 after yolk sac-implantation of NB-1 (**g**–**j**) and IMR-32 (**k**–**n**)) cells. Dotted lines are drawn according to Response Evaluation Criteria in Solid Tumors (RECIST) 1.1 adopted for zebrafish tumors, to visualize the best response: progressive disease (PD), at least a 20% increase in tumor volume; partial response (PR), at least a 30% decrease in tumor volume; each bar reflects one individual xenograft. The following treatments were applied: APR-246: 50 µM; vorinostat: 15 µM and combination.

Despite the generally high responsiveness, our analysis revealed that about 10% of neuroblastoma models were predicted to be less responsive to APR-246. As increased ROS levels augment the effect of APR-246 treatment, we assessed whether we could sensitize low-responsive cells to APR-246 treatment by ROS induction, e.g., by treatment with HDAC inhibitors. HDAC inhibitors are known ROS inducers (reviewed in [59]). We pretreated with 0.5 µM vorinostat for 72 h before addition of 20 µM APR-246 for 24 h and measured cell viability and cell death via trypan blue exclusion assay (Figure 4a–d). Consistent with our hypothesis, both compounds alone showed only minor effects, whereas the combination resulted in a significant reduction in the viable cell number and a significant increase in dead cells in APR-246 low-responsive SK-N-AS and SK-N-BE(2)-C cells.

Similar results were obtained with the HDACis abexinostat, romidepsin and Panobinostat in SK-N-AS, SK-N-BE(2)-C and IMR-32 cells (Table 5). For less responsive SK-N-AS cells at 72 h, co-treatment with HDACi vorinostat resulted in a HSA synergy score of 12.17, which indicated a synergistic effect of the drug combination (Figure 4e,f)). In line with our hypothesis, HDACi treatment increased ROS levels in IMR-32 cells and less responsive SK-N-AS cells had a lower basal ROS level compared to responsive IMR-32 cells (Figure S5).

To test the combination of the HDAC inhibitor and APR-246 in vivo, we used zebrafish embryo neuroblastoma xenograft models of NB-1 and IMR-32 cells. Fluorescently labeled tumor cells were transplanted into the yolk sack on day two post fertilization. Treatment with 50 µM APR-246, 1.5 µM vorinostat, solvent control and the combination was started on day three post fertilization. In both models, decreased tumor growth was observed under treatment with single compounds. However, in both cases the combination was more potent, underlining the effectiveness of combining HDAC inhibition and APR-246 treatment in neuroblastoma models (Figure 4g–n). Taken together, these results demonstrate that combining HDAC inhibition with ROS induction via APR-246 is effective in the treatment of neuroblastoma in vitro and in vivo.

## 4. Discussion

We studied APR-246 as a novel targeted treatment option for pediatric tumors of the nervous system and found neuroblastoma cell lines to be particularly responsive. We established a predictive genetic signature (log2*CORO1A* + log2*RHOH* + log2*CXCR4* + log2*PUM2* + log2*TRAF3IP3* + log2*KDM2B* + log2*PDE7A* + log2*ARHGAP9*)/(log2*PLS3* + log2*RHBDF1* + log2*CLDN1* + log2*SLC7A11* + log2*FAM114A1*), proposed a novel combinational treatment strategy for neuroblastoma, and validated the cell culture results in the in vivo zebrafish xenotransplantation model. Our results demonstrate that APR-246 is a promising drug candidate for the treatment of neuroblastoma.

The first study to investigate APR-246 in the field of pediatric oncology was conducted using Ewing sarcoma models and was one of the first studies to call p53 reactivation as the main and only mode of action into question [55]. Similar observations were made in a recent study on neuroblastoma. It was stated that APR-246 mainly acts via elevating the ROS level and inhibiting glutathione in this entity. The intracellular amount of glutathione was hypothesized to be modified by *MYCN* amplification and *TP53* mutation status. Notably, the neuroblastoma models used responded extraordinarily well to the treatment [60].

Whereas a relevant number of studies on tumor entities and clinical studies in adults revealed promising effects of APR-246, to our knowledge only a few studies have focused directly on the responsiveness of pediatric entities, including Ewing sarcoma, ALL, diffuse intrinsic pontine glioma, and neuroblastoma [55,60,61,62]. Recently, the combination of the HDACi Panobinostat and APR-246 has been described to be beneficial in the treatment of glioblastoma cell lines [63]. However, for many entities, the main mode of action of APR-246 remained unclear, predictive biomarkers are needed, and targeted treatment combination partners are missing.

In line with previous reports, we demonstrated that APR-246 responsiveness in pediatric tumors of the nervous system is independent of the *TP53* mutation status [60]. We validated a ROS mediated mode of action in neuroblastoma and demonstrated a predictive value of *SLC7A11* expression for discriminating responsive from less responsive entities. Using *SLC7A11* as a predictive entity marker, we identified ALL and NHL to be very responsive to PRIMA-1/APR-246 treatment. Furthermore, we determined a predictive genetic signature using the CCLE and challenged it with ten randomly picked pediatric cell line models. *YAP1* discriminated most significantly between responders and low-responders. *YAP1* and the genes from the identified signature may help to identify a novel APR-246 biomarker in other entities in future studies. Interestingly, *YAP1* is part of the genetic signature that discriminates between mesenchymal and adrenergic differentiation states in neuroblastoma [64,65]. In line with this, mesenchymal SH-EP and SK-N-AS cells were shown to be less responsive to APR-246, while adrenergic models, such as IMR-32 and SH-SY5Y, were responsive. Whether the neuroblastoma differentiation state is a possible response predictor remains to be further investigated.

Oxidative stress and ROS metabolism have been repeatedly described in the context of drug resistance [66,67,68]. We consistently found that ROS metabolism is a potential Achilles’ heel in acquired drug resistance of neuroblastoma models and highlight the possible use of APR-246 in the targeted treatment of vulnerabilities that are a consequence of chemoresistance. Due to its pharmacokinetics with unusually high plasma concentrations of up to 280 µM and the described mode of action (inhibiting glutathione and thus inducing ROS) APR-246 constitutes a promising second punch in the one-two punch model. As a first punch, that is supposed to elevate basal ROS levels and thereby increase sensitivity, we applied vorinostat, an HDACi, which was previously described to induce ROS in melanoma [21]. The combination was effective in sensitizing neuroblastoma models and acted synergistically with APR-246. Screening other broad-spectrum HDACis demonstrated that this effect is not vorinostat-exclusive. Identifying the most promising HDACis and investigating selective HDACis to minimize side effects are important future research perspectives.

One restriction of our study is the generally altered ROS metabolism in cell culture models and the question as to how the results can be translated into a clinical context (reviewed in Reference [69]). To address this question, we compared *SLC7A11* expressions in cell lines (CCLE) to *SLC7A11* expressions in patients (INFORM) and found that the same entities express *SLC7A11* low or high in vivo and in vitro. As in vivo metabolism might also alter treatment responses, we tested the HDACi/APR-246 combination in a zebrafish xenograft model and found that it was highly effective. Auspiciously, Wang et al. used a ROS inducing treatment strategy effectively in a patient, highlighting the seminal potential of this approach [21].

Our results and these other preliminary reports underline the possible and promising use of an ROS-inducing treatment strategy for neuroblastoma patients.

## 5. Conclusions

Our study highlights that ROS induction is a promising novel treatment strategy for neuroblastoma. We established a genetic signature that improves current predictiveness to APR-246 and will help guide future studies. To our knowledge, this is the first report to apply APR-246 and HDACi for the targeted treatment of neuroblastoma. Additionally, we are the first to provide in vivo data for the effective APR-246 treatment of neuroblastoma models.

## Figures and Tables

**Table 1:** Chemicals, reagents and diluents used.

Name	Company	Stock Conc., Diluent
APR-246	Selleck Chemicals, Munich, Germany	50 mM, DMSO
doxorubicin	Biozol, Eching, Germany	10 mM, H_2_O
vincristine	Enzo Life Sciences, Lörrach, Germany	10 mg/mL, DMSO
vorinostat	Selleck Chemicals, Munich, Germany	100 mM, DMSO
entinostat	Biomol GmbH, Hamburg, Germany	10 mM, DMSO
abexinostat	Selleck Chemicals, Munich, Germany	10 mM, DMSO
Panobinostat	Cayman Chemical, Hamburg, Germany	0.5 mM, DMSO
romidepsin	Biovision Inc., Milpitas, CA, USA	1 mM, DMSO
H_2_O_2_	Sigma-Aldrich, Munich, Germany	30% *w/w* H_2_O
DMSO	Sigma-Aldrich, Munich, Germany	-

**Table 2:** Sensitivity to APR-246 in five different pediatric tumor cell models of the nervous system, measured via metabolic activity (CTG).

Cell Line	Entity/*TP53* Status	IC50/95% CI [µM]
SJ-GBM2	pHGG ^1^/mut, p.R273C	71.8/69.3–74.4
SF188	pHGG/mut, p.G266E	43.9/39.4–48.8
KNS-42	pHGG/mut, p.R342 *^,2^	27.8/22.8–33.7
SK-N-BE(2)-C	neuroblastoma/mut, p.C135F	24.7/22.0–27.7
IMR-32 NIH-3T3 C5-GL (*TP53* ko) NIH-3T3 Δ6RT (*TP53* wt)	neuroblastoma/wt murine embryo fibroblasts murine embryo fibroblasts	4.0/3.2–4.8 18.4/15.8–21.3 18.7/17.6–19.9

^1^ pediatric high-grade glioma. ^2^ nonsense mutation (*) affecting the tetramerization domain, not the DNA binding domain of p53.

**Table 3:** Sensitivity to APR-246 in seven different models.

Cell Line	Entity	IC50/95% CI [µM]	*SLC7A11* Expression ^3^
Kelly	Neuroblastoma	8.48/6.8–10.0	1.43
NB-1	Neuroblastoma	4.83/4.22–5.42	1.35
SH-EP	Neuroblastoma	44.5/40.1–49.5	-
SK-N-FI	Neuroblastoma	9.21/8.02–10.1	0.88
NALM-6	ALL ^1^	4.56/3.09–6.85	2.51
REH	ALL ^1^	4.39/4.0–4.84	1.41
HuT 78	NHL ^2^	9.47/8.23–10.74	1.45

^1^ acute lymphoblastic leukemia. ^2^ non-Hodgkin lymphoma. ^3^ Log2 values, derived from CCLE, SH-EP are not included in CCLE.

**Table 4:** Biomarker validation in ten randomly picked samples (>1.0 predicts sensitivity; sensitivity is defined as IC50 < 20 µM).

Model Type	Name/Entity	Biomarker	IC50/95% CI [µM]
Established cell lines	SK-N-AS/neuroblastoma	1.03	35.1/29.2–43.1
	SIMA/neuroblastoma	1.80	2.04/1.74–2.4
	SH-SY5Y/neuroblastoma	1.68	19.7/16.8–22.4
	KNS-42/pHGG	0.90	27.8/22.8–33.7
	Jurkat/ALL	3.20	1.46/1.38–1.54
	JeKo-1/NHL	5.78	1.44/1.32–1.58
INFORM long-term cultures INFORM-PDX long-term culture	INF_R_1288_LTC/rhabdoid tumor	1.09	19.7/14.9–25.8
	INF_R_1467_LTC/soft tissue sarcoma	1.58	18.4/16.9–20.0
	INF_R_1490_LTC/osteosarcoma	1.19	28.7/25.5–32.0
	INF_R_1632_PDX_LTC/neuroblastoma	3.66	12.2/8.7–16.6

**Table 5:** HDACi screening for abexinostat, romidepsin and Panobinostat in three neuroblastoma cell line models.

Cell Line	HDAC Inhibitor	IC50 [nM]	95% CI [nM]	+ APR-246 [µM]	IC50 [nM]	95% CI [nM]
SK-N-AS	abexinostat	46.3	29.0–69.8	20	15.4	9.7–23.8
	romidepsin	5.7	4.9–6.6	20	1.8	1.5–2.2
	Panobinostat	2.2	1.6–2.9	20	1.0	0.6–1.4
SK-N-BE(2)-C	abexinostat	172.1	142.2–207.5	10	37.5	22.5–58.9
	romidepsin	26.9	15.2–140.9	10	9.9	3.6–52.9
	Panobinostat	40.2	29.4–54.6	20	3.1	0.3–12.2
IMR-32	abexinostat	100.0	56.3–169.7	2	30.4	6.6–48.9
	romidepsin	0.73	0.64–0.83	1	0.24	0.13–0.33
	Panobinostat	35.6	29.0–43.7	2	13.9	11.0–17.2

## Data Availability

The results published here are fully or partially based upon data generated by the Cancer Target Discovery and Development (CTD^2^) Network (https://ocg.cancer.gov/programs/ctd2/data-portal, accessed on 5 January 2021) established by the National Cancer Institute’s Office of Cancer Genomics. R2 (http://R2.amc.nl, accessed on 5 January 2021) and depmap portal (https://depmap.org/portal/, accessed on 5 January 2021) were used for in silico analysis. The CCLE datasets are available at the CCLE portal (www.broadinstitute.org/ccle, accessed on 5 January 2021).

## Supplementary Materials

The following are available online at https://www.mdpi.com/article/10.3390/cancers13174476/s1, Figure S1: *TP53* mutation status and PRIMA-1 responsiveness in pediatric entities. Figure S2: APR-246 is not restoring p53 pathway activity in neuroblastoma models IMR-32 (*TP53* wt) and SK-N-BE(2)-C (*TP53*mut, p.C135F). Figure S3: APR-246 is not restoring p53 pathway activity in *TP53* mutant pediatric high-grade glioma models SJ-GBM2 (*TP53*mut p.R273C) and SF188 (*TP53*mut, p.G266E). Figure S4: PRIMA-1 sensitivity and *SLC7A11* expression correlation for Cancer Cell Line Encyclopedia and CTD2 portal cancer entities. Figure S5: SK-N-AS has significantly lower basal ROS level compared to IMR-32 and vorinostat induces ROS in neuroblastoma cell line model.
