# Supplementary material for: Combining APR-246 and HDAC-Inhibitors: A Novel Targeted Treatment Option for Neuroblastoma
Source: Cancers (Basel). 2021 Sep 5;13(17):4476. doi: 10.3390/cancers13174476 (PMC8431508; doi:10.3390/cancers13174476)
Supplement: Supplementary file 1 [file cancers-13-04476-s001.zip › cancers-1366749-supplementary.pdf]

cancers
MDPI

Supplementary material

Combining APR-246 and HDAC-Inhibitors: A Novel Targeted Treatment Option for Neuroblastoma

Michael Müller, Lisa Rösch, Sara Najafi, Charlotte Gatzweiler, Johannes Ridinger, Xenia F. Gerloff, David T. W. Jone, Jochen Baßler, Sina Kreth, Sabine Hartlieb, Karen Frese, Benjamin Meder, Frank Westermann, Till Milde, Heike Peterziel, Olaf Witt and Ina Oehme

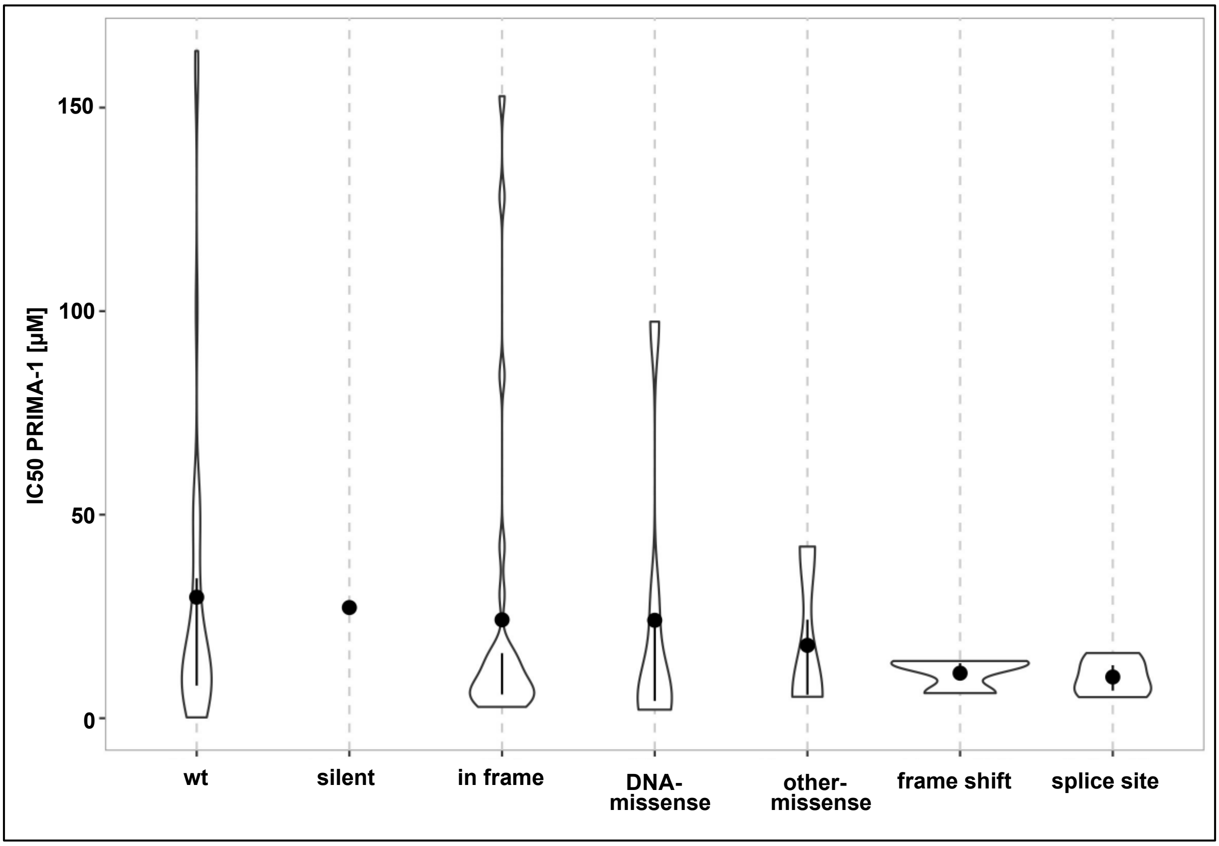

Figure S1. *TP53* mutation status and PRIMA-1 responsiveness in pediatric entities. PRIMA-1 sensitivity data filtered for pediatric entities (age < 18 when cell line was derived from patient, entities medulloblastoma and neuroblastoma were added if age was unknown, samples: n=73) separated upon *TP53* mutation status. Data derived from Cancer Cell Line Encyclopedia and CTD[2] portal.

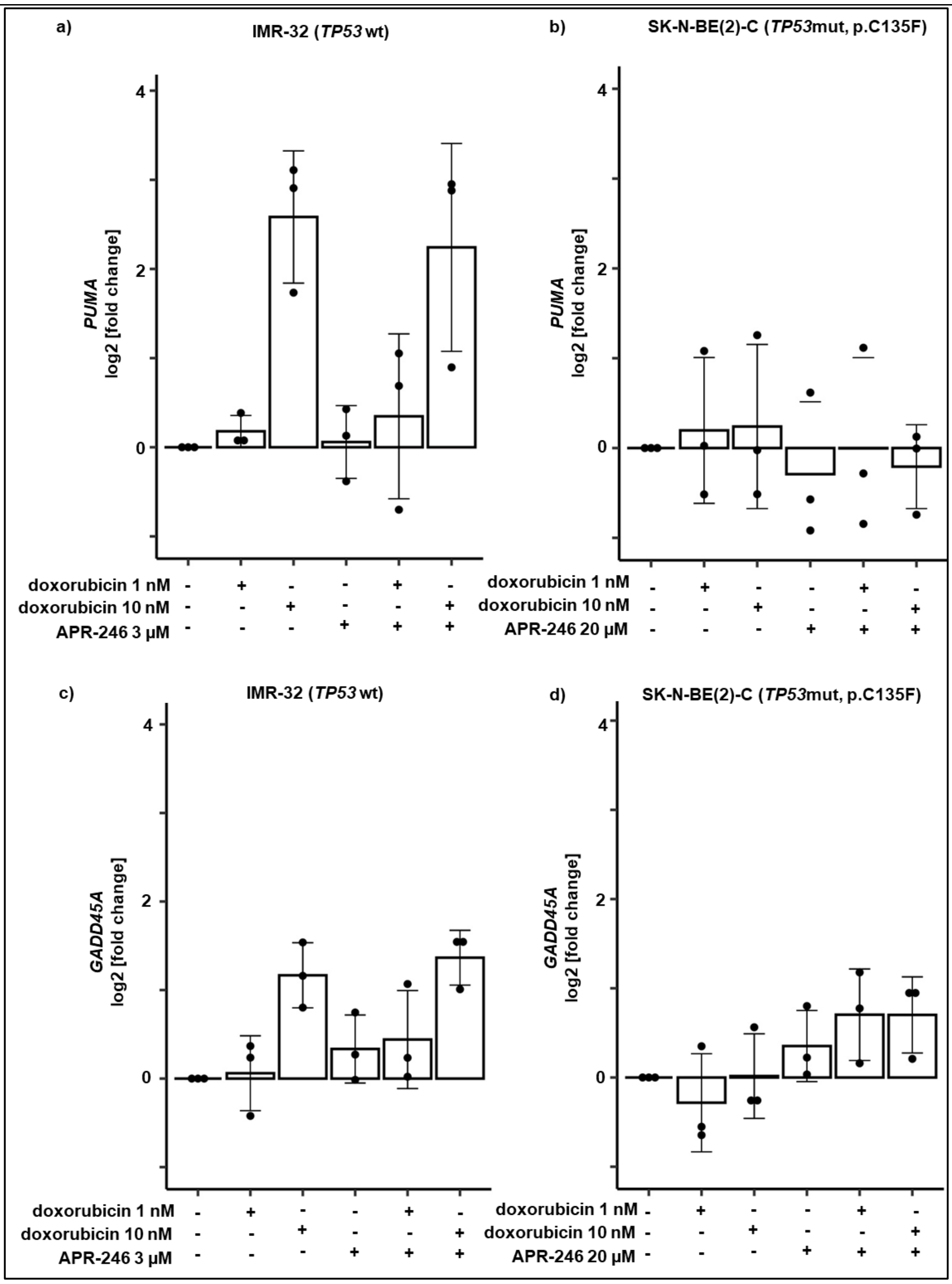

Figure S2. APR-246 is not restoring p53 pathway activity in neuroblastoma models IMR-32 (*TP53* wt) and SK-N-BE(2)-C (*TP53mut* p.C135F). RT-PCR measurement of *TP53* pathway activation. *PUMA* (IMR-32: (**a**); SK-N-BE(2)-C: (**b**) and *GADD45A* (IMR-32: (**c**); SK-N-BE(2)-C: (**d**)) expression are depicted as log2 transformed fold change to solvent control. Treatment was applied for 24 h with doxorubicin or APR-246, alone or in combination as indicated.

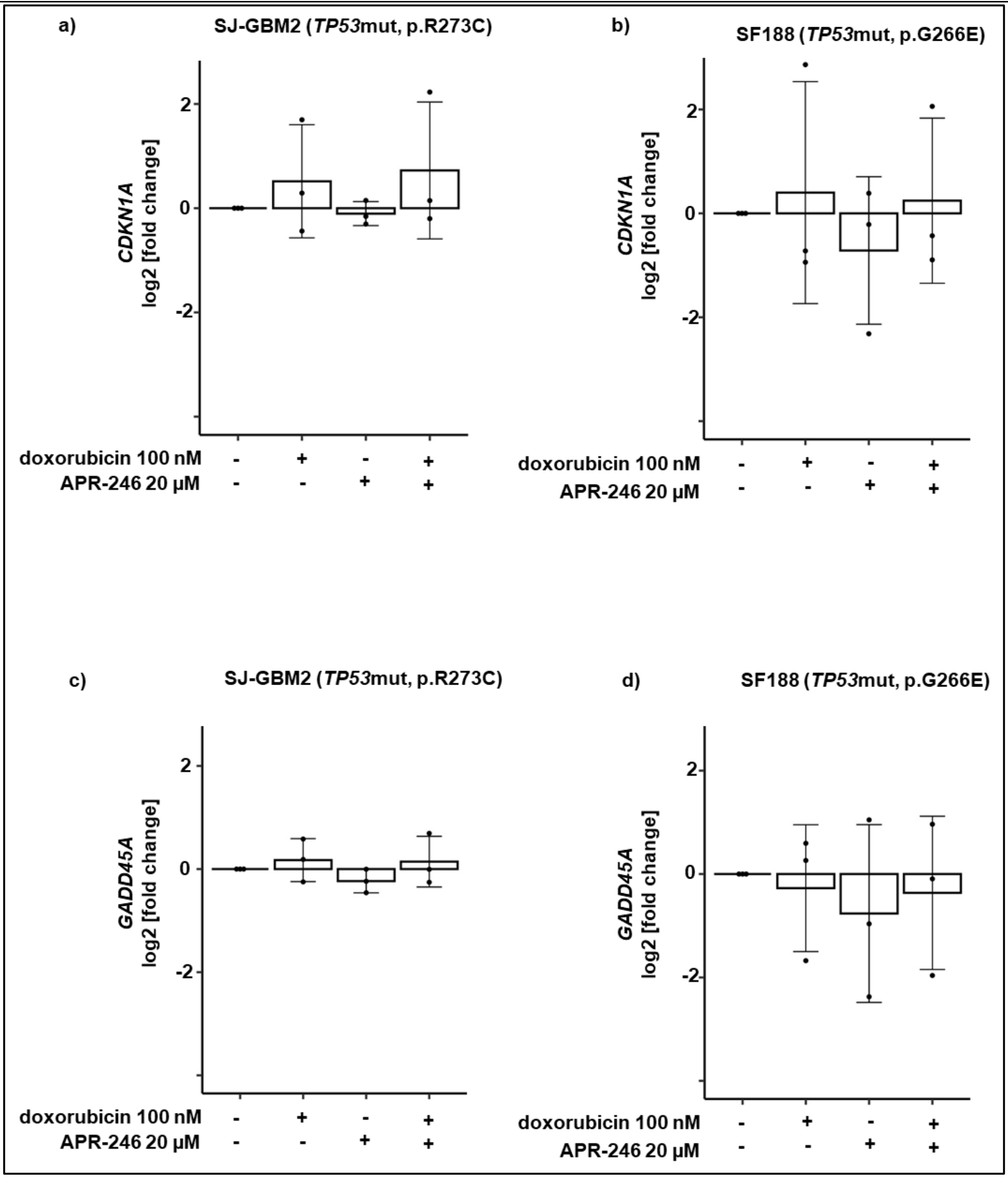

Figure S3. APR-246 is not restoring p53 pathway activity in *TP53* mutant pediatric high-grade glioma models SJ-GBM2 (*TP53mut* p.R273C) and SF188 (*TP53mut* p.G266E). RT-PCR measurement of p53 pathway activation. *CDKN1A* (SJ-GBM2: (**a**); SF188: (**b**)) and *GADD45A* (SJ-GBM2: (**c**); SF188: (**d**)) expression are depicted as log2 transformed fold change to solvent control. Treatment was applied for 24 h with doxorubicin or APR-246, alone or in combination as indicated. mut, mutant.

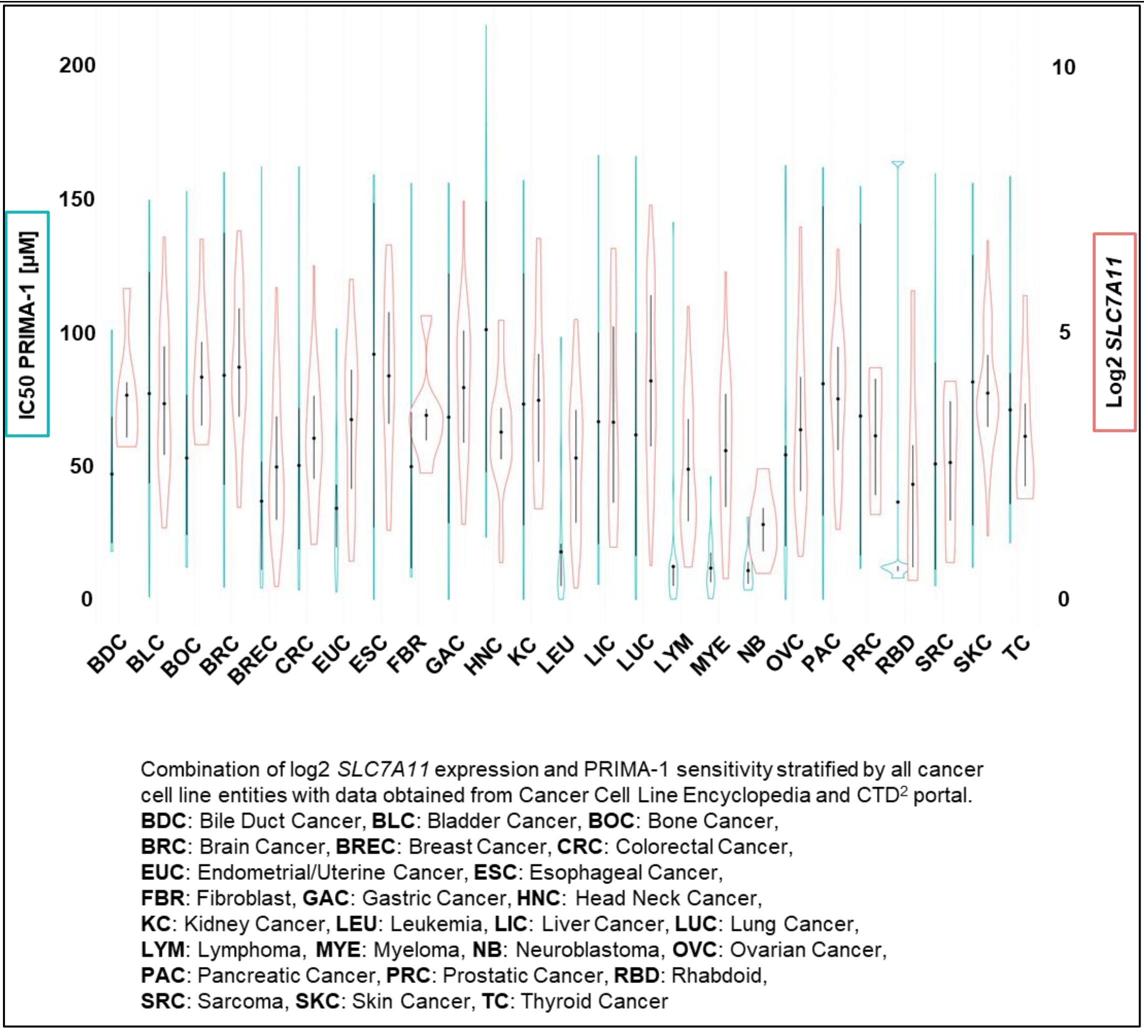

Figure S4. PRIMA-1 sensitivity and *SLC7A11* expression correlation for Cancer Cell Line Encyclopedia and CTD^2 portal cancer entities. Combination of log2 *SLC7A11* expression and PRIMA-1 sensitivity stratified by all cancer cell line entities with data obtained from Cancer Cell Line Encyclopedia and CTD^2 portal. BDC: Bile Duct Cancer, BLC: Bladder Cancer, BOC: Bone Cancer, BRC: Brain Cancer, BREC: Breast Cancer, CRC: Colorectal Cancer, EUC: Endometrial/Uterine Cancer, ESC: Esophageal Cancer, FBR: Fibroblast, GAC: Gastric Cancer, HNC: Head Neck Cancer, KC: Kidney Cancer, LEU: Leukemia, LIC: Liver Cancer, LUC: Lung Cancer, LYM: Lymphoma, MYE: Myeloma, NB: Neuroblastoma, OVC: Ovarian Cancer, PAC: Pancreatic Cancer, PRC: Prostatic Cancer, RBD: Rhabdoid, SRC: Sarcoma, SKC: Skin Cancer, TC: Thyroid Cancer

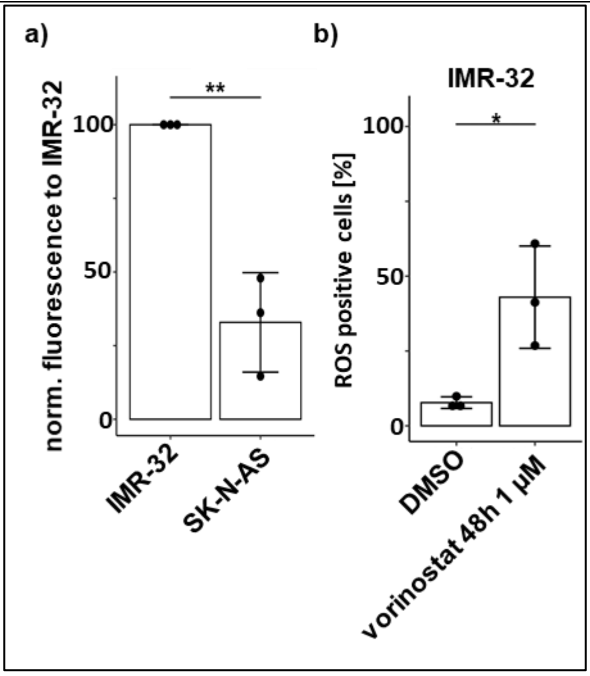

Figure S5. SK-N-AS has significantly lower basal ROS level compared to IMR-32 and vorinostat induces ROS in neuroblastoma cell line model. (**a**): Basal ROS level in IMR-32 and SK-N-AS detected after DCFDA staining by FACS, results were normalized to IMR-32 signal; (**b**): Increasing percentage of ROS positive IMR-32 cells after treatment with 1 µM vorinostat for 48 hours compared to solvent control (DMSO) detected after DCFDA staining by FACS.

Original Western Blots

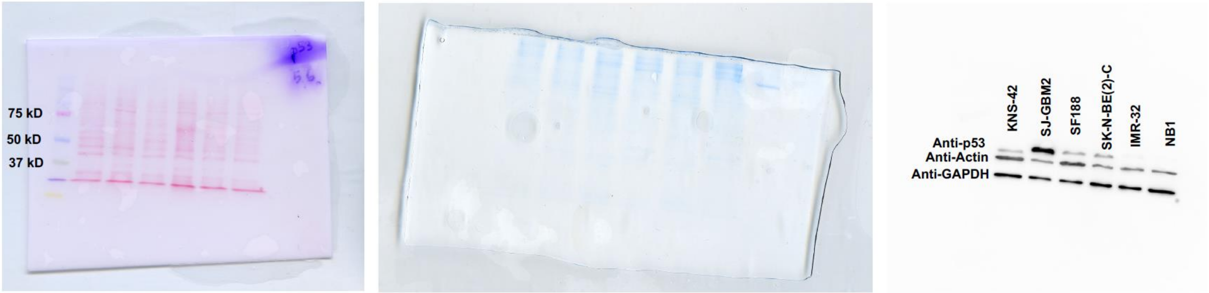

Lane Order: KNS-42, SJ-GBM2, SF188, SK-N-BE(2)-C, IMR-32, NB1 (NB1 not shown in publication
Antibody order (from top to bottom): anti-p53, anti-actin (not shown), GAPDH

R Script

```
#libraries####
library(readxl)
library(writexl)
library(tidyverse)
library(janitor)
setwd("~/Desktop")

#data####

#data is a tibble containing the samplenames, the drug response
#and the zscores of 171 Genes for 405 samples. The colnames correspond to the gene names
data <- read_xlsx("gene_expression_and_responsiveness.xlsx")
#gene_type lets you know which genes are classified as upregulated and which as downregulated
gene_type <- read_xlsx("gene_type.xlsx")
gene_names <- gene_type$gene_name
#test_projection functions####

#test_projection_single calculates how many of which types of errors were made
#by the projection and outputs these numbers in a named vector.
#the projection is made via the rule
#gene is responsive <-> zScore < 0 if gene is upregulated or zScore > 0 if gene is downregulated
test_projection_single <- function(gene_name, data){
  #get the gene's values
  index <- which(gene_names == gene_name)
  values <- data[,index+2]
  #make projection
  if(gene_type[index, 2] == "down"){
    projections <- values < 0
  } else{
    projections <- values > 0
  }
  #the following values store the row number in data of the projected or actual
  #responsive (=positive) and non-responsive (=negative) celllines
  projected_positives <- which(projections)
  projected_negatives <- which(!projections)
  actual_positives <- which(data$sensitivity)
  actual_negatives <- which(!data$sensitivity)
  #the following values count the different types of errors/successes of the projection
  num_true_positives <- sum(projected_positives %in% actual_positives)
  num_true_negatives <- sum(projected_negatives %in% actual_negatives)
  num_false_positives <- sum(projected_positives %in% actual_negatives)
  num_false_negatives <- sum(projected_negatives %in% actual_positives)
  result <- c("true_positives" = num_true_positives, "true_negatives" = num_true_negatives,
              "false_positives" = num_false_positives, "false_negatives" = num_false_negatives)
  dim(projections) <- NULL
  projection_tib <- tibble(data$samplenames,projections)
  colnames(projection_tib) <- c("samplenames", paste0("projection_based_on_", gene_name))
  return(result)
}
```

```
#group is a vector of gene names test_projection_group returns the analysis in the same way as test_projection_single
#the projection is made via the rule:
#responsive <-> sum of the downregulated Gene's values > 0 and sum of the upregulated Gene's values < 0
#if there are only upregulated Genes, only that part of the rule is used, same goes for the downregulated Genes.
test_projection_group <- function(group, data){
  size <- length(group)
  indices <- numeric(size)
  for(i in 1:size){
    indices[i] <- which(gene_names == group[i])
  }
  types <- gene_type[indices, 2]
  indices_down <- indices[which(types == "down")]
  indices_up <- indices[which(types == "up")]
  values_down <- data[ ,indices_down +2, drop = FALSE]
  values_down %>% mutate("sum" = rowSums(.)) -> values_down
  values_up <- data[, indices_up +2, drop = FALSE]
  values_up %>% mutate("sum" = rowSums(.)) -> values_up
  #make projection
  if(length(indices_up) == 0) {
    projections <- values_down$sum < 0
  } else if (length(indices_down) == 0){
    projections <- values_up$sum > 0
  } else{
    projections <- values_down$sum < 0 & values_up$sum > 0
  }
  #same as above
  projected_positives <- which(projections)
  projected_negatives <- which(!projections)
  actual_positives <- which(data$sensitivity)
  actual_negatives <- which(!data$sensitivity)
  #the following values count the different types of errors/successes of the projection
  num_true_positives <- sum(projected_positives %in% actual_positives)
  num_true_negatives <- sum(projected_negatives %in% actual_negatives)
  num_false_positives <- sum(projected_positives %in% actual_negatives)
  num_false_negatives <- sum(projected_negatives %in% actual_positives)
  result <- c("true_positives" = num_true_positives, "true_negatives" = num_true_negatives,
            "false_positives" = num_false_positives, "false_negatives" = num_false_negatives)
  return(result)
}

#analysis#####
n <- length(gene_names)
#make empty table for storing the results
single_gene_analysis <- tibble("gene_name" = gene_names, "true_positives" = numeric(n), "true_negatives" = nu-
meric(n),
                        "false_positives" = numeric(n), "false_negatives" = numeric(n))
#iterate through genes and perform the individual analysis
for (i in 1:n){
  result <- test_projection_single(gene_names[i], data)
  single_gene_analysis[i, 2:5] <- t(result)
}
```

```
single_gene_analysis %>% mutate("sum_of_errors" = false_positives + false_negatives) %>%
  mutate("percent_of_errors" = sum_of_errors/405, "sensitivity" = true_positives/(true_positives +false_negatives),
         "specificity" = true_negatives/(true_negatives + false_positives))-> single_gene_analysis

write_xlsx(single_gene_analysis, "single_gene_full_analysis.xlsx")



set1 <- filter(gene_type, type == "up")$gene_name
set2 <- rbind(arrange(single_gene_analysis, desc(sensitivity))[1:20, 1],
              arrange(single_gene_analysis, desc(specificity))[1:20, 1])
set3 <- filter(gene_type, type =="down")$gene_name

#analyse all sets of all sizes of upregulated genes
gene_set <- unlist(set1)
names(gene_set) <- NULL
combinations <- Map(combn, list(gene_set), seq_along(gene_set), simplify = FALSE)
combinations <- unlist(combinations, recursive = FALSE)
combinations <- lapply(combinations, function(x) {names(x) <- NULL; return(x)})
combinations_string <- sapply(combinations, function(x){str_c(x, collapse = ", ")})

n <- length(combinations)
multiple_genes_analysis <- tibble("gene_names" = combinations_string, "true_positives" = numeric(n), "true_nega-
tives" = numeric(n),
                        "false_positives" = numeric(n), "false_negatives" = numeric(n))
for(i in 1:n){
  if(i<length(gene_set)+1){
    gene_name <- combinations[[i]]
    result <- test_projection_single(gene_name, data)[[1]]
    multiple_genes_analysis[i, 2:5] <- t(result)
  }else{
    active_group <- combinations[[i]]
    result <- test_projection_group(active_group, data)
    multiple_genes_analysis[i, 2:5] <- t(result)
  }
}

multiple_genes_analysis %>% mutate("sum_of_errors" = false_positives + false_negatives) %>%
  mutate("percent_of_errors" = sum_of_errors/405,  "sensitivity" = true_positives/(true_positives + false_negatives),
         "specificity" = true_negatives/(true_negatives + false_positives))-> multiple_genes_analysis

write_xlsx(multiple_genes_analysis, "upregulated_genes_full_analysis.xlsx")

#analyse sets of five genes from set2
gene_set <- unlist(set2)
names(gene_set) <- NULL
combinations <- combn(gene_set,5)
combinations <- lapply(seq_len(ncol(combinations)), function(i) combinations[,i])
combinations_string <- sapply(combinations, function(x){str_c(x, collapse = ", ")})

n <- length(combinations)
```

```
multiple_genes_analysis <- tibble("gene_names" = combinations_string, "true_positives" = numeric(n), "true_nega-
tives" = numeric(n),
                          "false_positives" = numeric(n), "false_negatives" = numeric(n))
for(i in 1:n){
  active_group <- combinations[[i]]
  result <- test_projection_group(active_group, data)
  multiple_genes_analysis[i, 2:5] <- t(result)
}

multiple_genes_analysis %>% mutate("sum_of_errors" = false_positives + false_negatives) %>%
  mutate("percent_of_errors" = sum_of_errors/405,  "sensitivity" = true_positives/(true_positives + false_negatives),
       "specificity" = true_negatives/(true_negatives + false_positives))-> multiple_genes_analysis

write_xlsx(multiple_genes_analysis, "20_best_sens_and_20_best_spec_groups_of_five_analysis.xlsx")
```
